# Enhancing Infectious Disease Preparedness in Rome: The Lazio Regional Network During the 2025 Jubilee

**DOI:** 10.3390/tropicalmed11080211

**Published:** 2026-07-26

**Authors:** Gaetano Maffongelli, Andrea Bongiovanni, Alessandra D’Abramo, Erica Binetti, Laura Scorzolini, Claudia Palazzolo, Gabriella De Carli, Alessandro Agresta, Francesco Vairo, Emanuele Nicastri

**Affiliations:** National Institute for Infectious Diseases ‘Lazzaro Spallanzani’ IRCCS, Via Portuense 292, 00149 Rome, Italy; gaetano.maffongelli@inmi.it (G.M.); alessandra.dabramo@inmi.it (A.D.); erica.binetti@inmi.it (E.B.); laura.scorzolini@inmi.it (L.S.); claudia.palazzolo@inmi.it (C.P.); gabriella.decarli@inmi.it (G.D.C.); alessandro.agresta@inmi.it (A.A.); francesco.vairo@inmi.it (F.V.); emanuele.nicastri@inmi.it (E.N.)

**Keywords:** mass gathering, surveillance, preparedness, Hub-and-Spoke model, telemedicine

## Abstract

In 2025, Rome hosted the Catholic Jubilee, recording over 33 million cumulative pilgrim attendances while simultaneously facing the death of Pope Francis, the election of Pope Leo XIV, and a regional West Nile Virus (WNV) outbreak. This commentary describes the Lazio Region’s preparedness strategy, based on adapting an existing Hub-and-Spoke infectious diseases network integrating surveillance with Regional Service for Epidemiology, Surveillance and Control of Infectious Diseases (SERESMI) laboratory support, telemedicine, and clinical management. Preparatory measures included an additional Hub, updated syndromic pathways, multidisciplinary coordination, and enhanced surveillance. Emergency department visits increased by 14.8%, while the proportion of infectious syndromes remained stable. This experience highlights the potential value of strengthening existing healthcare networks for mass gathering preparedness.

## 1. Introduction

Mass gatherings (MGs) are events attended by a sufficient number of people to strain the planning and response capacities of local, regional, or national systems [1]. The World Health Organization (WHO) has highlighted that large-scale events can rapidly overwhelm healthcare infrastructures and therefore require targeted preparedness and response strategies [2]. Such settings facilitate the transmission of infectious diseases, particularly respiratory and gastrointestinal infections, because of the high density of participants [3]. To strengthen preparedness, the Lazio Region adopted a Hub-and-Spoke model for its infectious diseases network, integrating specialist referral centres, peripheral hospitals, telemedicine, laboratory support, and epidemiological surveillance. Widely used for time-dependent conditions such as stroke, myocardial infarction, and trauma [4], this organizational model reflects the principles of network governance, whereby autonomous organizations coordinate through shared protocols and collaborative decision-making to improve preparedness and response capacity [5]. This commentary describes how this organizational model was adapted to strengthen preparedness for the 2025 Jubi-lee through the integration of epidemiological surveillance, laboratory diagnostics, clinical management, and hospital referral pathways.

## 2. Data Sources

Data on telemedicine activity were extracted from the LazioAdvice regional platform, which is used to manage teleconsultations within the Lazio Region’s Hub-and-Spoke Network for Infectious Diseases. The platform records all teleconsultation requests, including information on the requesting facility, the reference Hub, the date of the request, and key clinical and care-related characteristics.

Data on reportable infectious diseases were obtained from the national surveillance system PREMAL, which is used for the mandatory reporting of infectious diseases by the Prevention Departments of the Local Health Authorities.

Syndromic surveillance data on emergency department (ED) visits were extracted from the regional information system GIPSE, which collects data on visits to EDs in the Lazio Region. Visits are classified into 15 syndromes of public health interest using algorithms based on ICD-9-CM diagnostic codes. For each syndrome, the number of observed cases is compared with the expected number estimated using the Farrington Flexible algorithm, which is based on historical data and implemented using the surveillance package of the R software (version 4.3.3, R Foundation for Statistical Computing, Vienna, Austria), enabling the early identification of abnormal increases in syndrome activity.

## 3. Preparing for the 2025 Catholic Jubilee

The Lazio Region, located in central Italy, includes Rome, the Italian capital, and has approximately 5.7 million inhabitants. Its role as the country’s administrative centre and host of Vatican City makes it a frequent setting for large international MGs [6].

Rome has a long-standing experience in managing public health challenges related to mass gatherings. During the Millennium Jubilee in 2000, approximately 26 million visitors attended the event. Pilgrims originated from diverse geographical regions, had relatively short stays (mean 2.5 days), and gathered in large crowds. In response, infectious disease surveillance in the Lazio Region was strengthened through a laboratory-based system targeting diseases with high epidemic potential, including foodborne infections and meningitis [7].

During the 2025 Jubilee, Rome recorded more than 33.5 million cumulative pilgrim attendances from 185 countries [8]. Concurrently, the Lazio Region managed the death and funeral of Pope Francis, the conclave, the election of Pope Leo XIV, and the first regional outbreak of West Nile virus. To address these challenges, the Regional Health Authority, together with the National Civil Protection Department, strengthened emergency preparedness through enhanced syndromic surveillance, expanded hospital surge capacity, and coordinated emergency planning [9]. Although the Regional Infectious Diseases Network predated the Jubilee, preparedness relied on the coordinated adaptation of existing capacities rather than the creation of new structures. Consistent with the principles of network governance [5] and health system resilience [10], infectious diseases (ID) units, EDs, laboratories, epidemiological surveillance, and telemedicine were integrated within a Hub-and-Spoke model to support coordinated clinical management and public health response.

## 4. Surge Capacity of the Lazio Infectious Diseases Network

**Figure 1:**
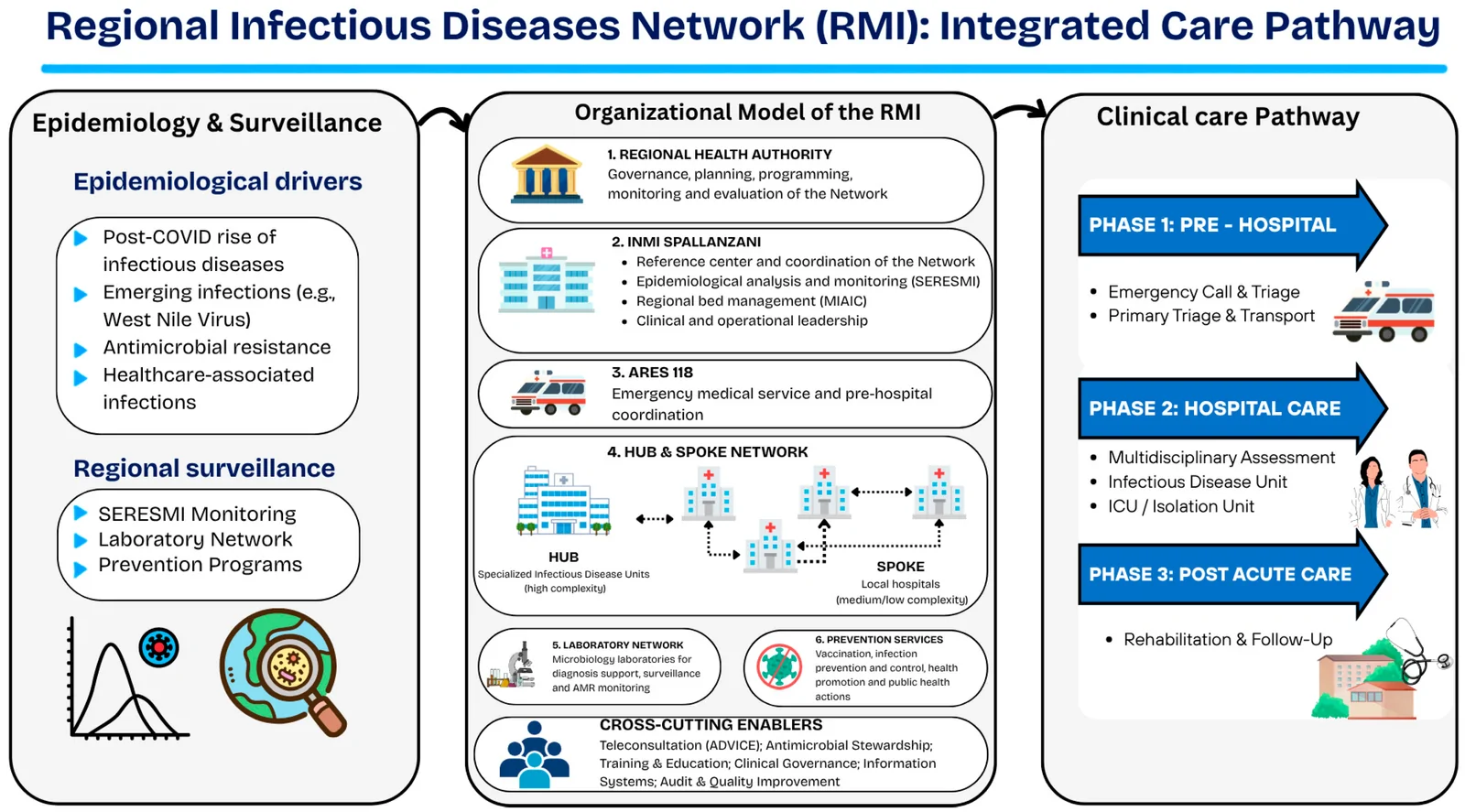
Organization of the Lazio Regional Infectious Diseases Network and integrated care pathway for ID preparedness and response.

Clinical surge capacity in the Lazio Region was reorganized to address the increased ID risk associated with the 2025 Jubilee. Since 2015, the Regional Health Authority has maintained a hospital-based Infectious Diseases Network organized according to a Hub-and-Spoke model integrating clinical care, surveillance, and prevention activities [11] (Figure 1). For the Jubilee, the network was strengthened through the designation of a fourth Hub, redistribution of referral pathways according to geographical catchment areas, inclusion of a pediatric IDs HUB, and revision of syndromic clinical pathways.

The network comprises four Hubs providing 24 h per day, every day specialist support, four intermediate Hubs operating 12 h per day, six days per week, and multiple spokes, mainly emergency departments without dedicated ID units. The Italian National Institute for Infectious Diseases “Lazzaro Spallanzani” provides technical and scientific support for network planning and clinical governance, while regional ID units act as referral centres and support spoke hospitals in the management of infectious syndromes, healthcare-associated infections, and antimicrobial stewardship [12].

Regular multidisciplinary meetings involving ID specialists, emergency physicians, microbiologists, public health professionals, and regional health authorities supported coordinated decision-making through continuous review of operational procedures, sharing of surveillance data and clinical experience, and timely updates of clinical pathways throughout the Jubilee. These meetings also provided a structured mechanism for rapid dissemination of updated case definitions, surveillance alerts, and organizational changes across the regional network. The multidisciplinary meetings also facilitated coordination with the broader regional emergency preparedness framework, including public health authorities and the National Civil Protection Department when required. Telemedicine represented a key component of the surge strategy. Established as a regional priority since 2019, the teleconsultation platform supported specialist decision-making across the network and had previously been used during the COVID-19 pandemic [11]. From 2023 onwards, referrals to ID units progressively increased (21% in 2023, 23% in 2024, and 27% in 2025). Although this trend is consistent with greater use of the regional network following the implementation of updated clinical pathways, alternative explanations, including changes in referral practices, case-mix, and organizational factors, cannot be excluded.

To standardize clinical management, syndromic assessment protocols were updated according to current evidence and international recommendations. Existing pathways for respiratory illness, neurological syndromes, fever in returning travellers, infectious exanthems, hepatitis, gastroenteritis, and sepsis were revised, while additional pathways for acquired immunodeficiency syndrome, high consequence infectious diseases, and gender based violence were introduced [12]. The protocols are available through the Spal-lanzani institutional platform (https://www.inmi.it/bedmanager/, accessed on 20 May 2026).

## 5. The Lazio Infectious Disease Surveillance and Response Plan

The Lazio Region developed a dedicated plan to strengthen ID surveillance and response before and during the Jubilee. The main objective was to strengthen passive surveillance, particularly for syndromes associated with airborne, foodborne and vector-borne transmission. The syndromic surveillance system, based on ED visits, was expanded and automated using the Farrington algorithm to rapidly detect unexpected increases in acute infectious disease presentations.

Epidemic intelligence activities continued through ongoing monitoring of international sources, including WHO and the European Centre for Disease Prevention and Control (ECDC), as well as through the Italian Network of ECDC Epidemic Intelligence from Open Sources (EIOS) event-based surveillance system. These activities were supported by an integrated digital platform that collected and analyzed data from established surveillance systems, enabled spatial and temporal analyses, and generated automated alerts for notifiable diseases. These initiatives supported the production of comprehensive weekly internal epidemiological reports to inform timely public health decision-making.

Before the Jubilee, priority diseases with outbreak potential were assessed using the ECDC rapid risk assessment methodology [13]. Healthcare staff also received targeted training to strengthen surveillance, case management, and response to infectious emergencies.

Passive surveillance of notifiable diseases showed an increase in reported cases, from 7150 in 2023 to 9802 in 2024 (+37.1%) and 9918 in 2025 (+1.2%). During the Jubilee year, this represented only a modest rise, lower than expected. Similarly, ED visits (Table 1) increased from 1,589,510 in 2023 to 1,824,878 in 2025 (+14.8%). Over the same period, the total number of infectious syndrome classifications increased from 291,158 in 2023 to 319,550 (+9.8%) in 2024 and 343,327 (+7.4%) in 2025. However, the proportion of infectious syndrome classifications relative to total ED visits remained substantially stable (18.32% in 2023, 19.04% in 2024 and 18.81% in 2025).

Respiratory syndromes represented the largest share of infectious syndrome classifications and remained proportionally stable over time despite increasing in absolute number (41.5–41.74%). No major changes were observed in the other syndromic groups, except for gastrointestinal syndromes, which showed an increase in both absolute counts and relative frequency, with bloody diarrhea increasing from 26,107 (8.97%) to 33,833 (9.85%) and gastroenteritis from 31,593 (10.85%) to 40,250 (11.72%). A more modest increase was also observed for sepsis or unexplained shock, from 0.97% to 1.49%.

Lazio, particularly the province of Latina, experienced its first autochthonous WNV outbreak, unrelated to the Jubilee. The outbreak was part of a broader national pattern, affecting multiple Italian provinces, including some with no previous documented WNV cases. In Lazio, 273 confirmed and 22 probable cases were reported, including 88 neuroinvasive and 175 febrile cases, along with 10 positive donors.

Although the WNV outbreak was epidemiologically unrelated to the Jubilee, it occurred while the enhanced preparedness system was fully operational. Consequently, the organizational measures implemented for the Jubilee—including strengthened coordination within the regional infectious diseases network, reinforced epidemiological surveillance, regular multidisciplinary coordination meetings, updated clinical pathways, and rapid communication between hospitals, laboratories, and public health authorities—also supported the regional response to this concurrent public health emergency.

A rapid regional public health response was implemented to contain the outbreak. Measures included strengthened entomological and veterinary surveillance for early detection of viral circulation, vector control with larvicides and adulticides, public risk communication, reinforced safety of blood and tissue donations, and training webinars for healthcare workers [14].

## 6. Conclusions

MGs such as the Jubilee require integrated preparedness strategies linking clinical care, laboratory diagnostics, epidemiological surveillance, and coordinated governance. Unlike the Hajj, where prolonged close contact among pilgrims is associated with a high burden of respiratory infections and exacerbations of chronic respiratory diseases [15], the 2025 Jubilee involved a geographically dispersed population, requiring preparedness for a broader spectrum of infectious threats, including vector-borne diseases. Although this study was not designed to formally evaluate the effectiveness of the implemented interventions, our experience suggests that strengthening existing infectious diseases networks, rather than creating temporary parallel structures, is a feasible and sustainable approach to mass gathering preparedness. Our experience also suggests that regular multidisciplinary coordination meetings involving Hub-and-Spoke centres, regional health authorities, and the Emergency Medical Service (ARES 118) facilitate timely information sharing, coordinated decision-making, and streamlined implementation of organizational measures. The integration of a Hub-and-Spoke model with multidisciplinary collaboration and telemedicine may therefore provide an organizational framework adaptable to other healthcare settings according to local resources and healthcare needs.

A further area of development concerns the gradual evolution of surveillance systems from a predominantly reactive approach towards models of proactive preparedness. The experience gained during the COVID-19 pandemic with wastewater surveillance has highlighted the potential for integrating complementary data sources with traditional epidemiological surveillance. On this basis, similar approaches are currently being implemented as part of the National Plans dedicated to the surveillance of antimicrobial resistance and respiratory pathogens with pandemic potential.

Finally, future research should complement descriptive reports with robust quasi-experimental designs, such as Interrupted Time Series or Difference-in-Differences analyses, to better evaluate the effectiveness, organizational impact, and sustainability of preparedness strategies.

This commentary has some limitations. Its descriptive design and the lack of comparator data do not allow causal inference regarding the impact of the implemented interventions. Patient-level outcomes and cost-effectiveness analyses were beyond the scope of this work. Future studies should incorporate comparative designs and standardized performance indicators to evaluate preparedness strategies more comprehensively.

The concurrent management of the Jubilee and the WNV outbreak illustrates the value of integrating clinical care, laboratory support, and epidemiological surveillance within a coordinated regional network. Future preparedness should build on existing healthcare infrastructures through regular multidisciplinary training, periodic revision of clinical pathways, and interoperable digital systems integrated into routine regional healthcare planning.

## Figures and Tables

**Table 1:** ED visits and syndromic classifications by year (2023–2025).

Syndrome	2023		2024				2025			
	N	% ^1^	N	% ^1^	Δ N (%)	Δ pp	N	% ^1^	Δ N (%)	Δ pp
Comatose state	740	0.25	665	0.21	−10.1	−0.05	538	0.15	−19.1	−0.06
Diarrhea with blood in stool without evidence of bleeding from other sites	26,107	8.96	30,119	9.43	15.4	0.47	33,833	9.85	12.3	0.43
Acute hemorrhagic syndrome	55,273	18.98	56,048	17.54	1.4	−1.44	56,983	16.60	1.7	−0.94
Fever with nonspecific rash	7310	2.51	9252	2.90	26.6	0.38	9884	2.88	6.8	−0.02
Fever with rash suggestive of exanthematous diseases	7760	2.67	10,196	3.19	31.4	0.53	10,602	3.09	4.0	−0.10
Gastroenteric syndrome without blood in stool	31,593	10.85	36,206	11.33	14.6	0.48	40,250	11.72	11.2	0.39
Acute jaundice syndrome	3307	1.14	3838	1.20	16.1	0.07	4213	1.23	9.8	0.03
Acute localized skin lesion	5262	1.81	6212	1.94	18.1	0.14	7128	2.08	14.7	0.13
Lymphadenitis with fever	1621	0.56	1960	0.61	20.9	0.06	2102	0.61	7.2	0.00
Unexplained death	120	0.04	149	0.05	24.2	0.01	156	0.05	4.7	0.00
Neurological syndrome	8066	2.77	7471	2.34	−7.4	−0.43	7846	2.28	5.0	−0.06
Peripheral neurological syndrome	4328	1.49	4678	1.46	8.1	−0.02	5220	1.52	11.6	0.06
Respiratory syndrome	120,838	41.50	132,710	41.53	9.8	0.03	143,298	41.74	8.0	0.21
Sepsis or unexplained shock	2830	0.97	4315	1.35	52.5	0.38	5116	1.49	18.6	0.14
Fever without localizing signs	16,003	5.50	15,731	4.92	−1.7	−0.57	16,158	4.71	2.7	−0.22
Infectious syndrome classifications	291,158	100.00	319,550	100.00	9.8	—	343,327	100.00	7.4	—

^1^ Percentages are calculated using the total number of infectious syndrome classifications as the denominator. A single ED visit may be assigned to more than one infectious syndrome classification.

## Data Availability

The data presented in this study are available on request from the corresponding author due to privacy and institutional restrictions.

## Abbreviations

The following abbreviations are used in this manuscript:

EBS	Event-Based Surveillance
ECDC	European Centre for Disease Prevention and Control
EDs	Emergency Departments
ID	Infectious Disease
MGs	Mass Gatherings
SERESMI	Regional Service for Surveillance and Control of Infectious Diseases
WHO	World Health Organization
WNV	West Nile Virus
